# Antitumor Effect of a Novel Spiro-Acridine Compound is Associated with Up-Regulation of Th1-Type Responses and Antiangiogenic Action

**DOI:** 10.3390/molecules25010029

**Published:** 2019-12-20

**Authors:** Daiana K. Frade Silva, Sâmia S. Duarte, Thaís M. H. Lisboa, Rafael C. Ferreira, Ana Luíza de O. Lopes, Deyse C. M. Carvalho, Sandra Rodrigues-Mascarenhas, Patricia Mirella da Silva, Miguel A. S. Pinheiro Segundo, Ricardo O. de Moura, Karina C. P. Medeiros, Marianna V. Sobral

**Affiliations:** 1 Post Graduation Program in Bioactive Natural and Synthetic Products, Federal University of Paraíba, João Pessoa, PB 58051-970, Brazil; daiana.frade@gmail.com (D.K.F.S.); samiasduarte@gmail.com (S.S.D.); thaismangeon@gmail.com (T.M.H.L.); rafaelcarlos@ltf.ufpb.br (R.C.F.); ana.lopes0407@gmail.com (A.L.d.O.L.); sandra@cbiotec.ufpb.br (S.R.-M.); 2 Multicenter Postgraduate Program in Physiological Sciences, Federal University of Paraíba, João Pessoa, PB 58051-970, Brazil; deysecmc@gmail.com; 3 Department of Molecular Biology, Federal University of Paraíba, João Pessoa, PB 58051-970, Brazil; mirella_dasilva@hotmail.com; 4 Post Graduation Program in Pharmaceutical Sciences, Federal University of Pernambuco, Recife, PE 50670-901, Brazil; miguelssegundo@gmail.com; 5 Drug Development and Synthesis Laboratory, Department of Pharmacy, State University of Paraíba, João Pessoa, PB 58070-450, Brazil; ricardo.olimpiodemoura@gmail.com; 6 Department of Morphology, Federal University of Rio Grande do Norte, Natal, RN 59078-970, Brazil; karinapm@yahoo.com; 7 Department of Pharmaceutical Sciences, Federal University of Paraiba, João Pessoa, PB 58051-970, Brazil

**Keywords:** spiro-acridine compound, angiogenesis, antitumor activity, immune response

## Abstract

Tumor cells have specific features, including angiogenesis induction, cell cycle dysregulation, and immune destruction evasion. By inducing a T helper type 2 (Th2) immune response, tumor cells may favor immune tolerance within the tumor, which allows progression of cancer growth. Drugs with potential antitumor activity are the spiro-acridines, which is a promising new class of acridine compounds. Herein, the novel spiro-acridine (*E*)-5′-oxo-1′-((3,4,5-trimethoxybenzylidene)amino)-1′,5′-dihydro-10*H*-spiro[acridine-9,2′-pyrrole]-4′-carbonitrile (AMTAC-17) was synthesized and tested for antitumor effects. Toxicity evaluation was performed in mice after acute treatment (2000 mg/kg, intraperitoneally, i.p.). The Ehrlich ascites carcinoma model was used to investigate the antitumor activity of AMTAC-17 (12.5, 25, or 50 mg/kg, i.p.) after seven days of treatment. Effects on the cell cycle, angiogenesis, and inflammatory responses were investigated. LD_50_ (lethal dose 50%) was estimated to be higher than 5000 mg/kg. AMTAC-17 reduced the Ehrlich tumor’s total viable cancer cells count and peritumoral micro-vessels density, and induced an increase in the sub-G1 peak. Additionally, there was an increase of Th1 cytokine profile levels (IL-1β, TNF-α, and IL-12). In conclusion, the spiro-acridine compound AMTAC-17 presents low toxicity, and its in vivo antitumor effect involves modulation of the immune system to a cytotoxic Th1 profile and a reduction of tumor angiogenesis.

## 1. Introduction

Cancer cells exhibit specific hallmarks including cell cycle dysregulation and angiogenesis induction, besides producing a tumor microenvironment where tumor cells are associated with bioactive molecules of inflammatory stromal cells to induce their proliferation, survival, and angiogenesis [1]. Cell cycle dysregulation is related with the maintenance of proliferative signaling and resistance to apoptosis [2]. In addition, angiogenesis induces the tumor growth by providing oxygen and nutrients as well as favoring metastasis [3].

Moreover, tumor cells can escape immune surveillance cells [4], promoting immune tolerance to the tumor by favoring the establishment of a Th2 immune response, expressing cytokines like IL-4, IL-10, and TGF-β [5]. Previous studies show that a sustained immune change from Th2 to Th1 profiles is critical for tumor cell death [6].

**Figure 1:**
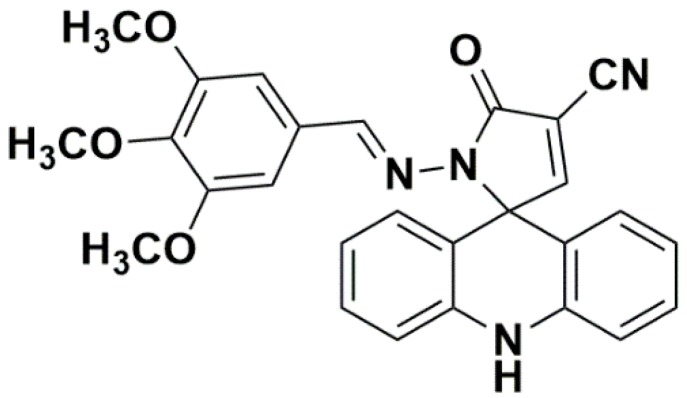
Structure of spiro-acridine compound (*E*)-5′-oxo-1′-((3,4,5-trimethoxybenzylidene) amino)-1′,5′-dihydro-10*H*-spiro[acridine-9,2′-pyrrole]-4′-carbonitrile (AMTAC-17).

Spiro-acridines are a promising new class of acridine derivatives [7], which have been obtained by spirocyclization reactions yielding a five-membered or six-membered spiro ring attached to acridine C-9 carbon [8]. These compounds have shown DNA binding capacity, inhibition of human topoisomerase IIα and tyrosinase enzyme, and antiproliferative activity against tumor cell lines [8,9,10]. Herein, we described the synthesis of a novel spiro-acridine compound, (*E*)-5′-oxo-1′-((3,4,5-trimethoxybenzylidene)amino)-1′,5′-dihydro-10*H*-spiro[acridine-9,2′-pyrrole]-4′-carbonitrile (AMTAC-17, Figure 1), and evaluated the mechanism of antitumor action on the Ehrlich ascites carcinoma model.

## 2. Results and Discussion

### 2.1. Chemistry

The compound AMTAC-17 was idealized from the results obtained by Almeida et al. (2016), who, in their previous studies, observed that the methoxylated spiro-acridine derivative in the benzylidenic ring (AMTAC-02) presented the best result of antiproliferative activity, besides toposimoresase IIα inhibitory activity, which is comparable to the amsacrine pattern. Thus, we believe that because AMTAC-02 has important ionizable chemical groups, it may possibly result in electrostatic attraction between the drug and biomolecular targets, or DNA/topoisomerase. Thus, we add two more methoxyl groups, both in the meta position and one in the para position in relation to the benzylidene ring. We analyze the effects of these additions. The synthesized compound presents its spectral data and physicochemical characteristics described below.

**Scheme 1:**
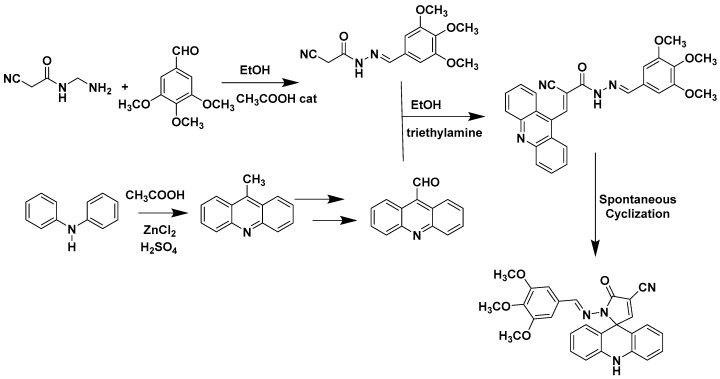
Synthetic route to obtain spiro-acridine derivatives (AMTAC-17).

The synthetic route used to obtain the spiro-acridine derivatives (Scheme 1) was parallel and convergent, where we will perform the synthesis of 2-cyano-*N*’-(3 4,5-trimethoxy-benzylidene) -acetohydrazide (JR-10) in parallel with the acridine aldehyde. To obtain the JR-10, we started from 2-cyano-acetohydrazide, which was condensed with 3,4,5-trimethoxy-benzaldehyde in ethanolic medium and molar equivalents acid catalytic at room temperature for 24 h. For the synthesis of acridine aldehyde, we start from diphenylamine, which undergoes a Frield Crafts acylation reaction followed by cyclization in acid medium. This leads to the 9-methylacridine, which, after successive oxidations, we obtain the 9-carboxyaldehyde-acridinic. To obtain the spiro-acridine derivatives, we started from the acridine aldehyde, which was condensed with the intermediate JR-10 in ethanolic and basic medium in molar equivalents at 78 °C for 6 h, which undergo spontaneous cyclization and leads to the final spiro-acridine derivatives.

### 2.2. Biological Evaluation

In order to determine safe doses to be used for in vivo pharmacological tests, the acute non-clinical toxicity assay was performed. No death was recorded after AMTAC-17 (2000 mg/kg) treatment, according to the OECD guideline. The LD_50_ was estimated to be higher than 5000 mg/kg, which suggests low acute toxicity in mice [11].

AMTAC-17 induced a significant reduction on total viable cancer cell count (46.47 ± 4.78 × 10^7^ cells, 61.82 ± 8.66 × 10^7^ cells, and 57.98 ± 5.27 × 10^7^ cells for 12.5, 25, and 50 mg/kg, respectively; *p* < 0.05) when compared to the tumor control group (135.5 ± 10.95 × 10^7^ cells). The AMTAC-17 12.5-mg/kg dose was chosen to study the mechanism of antitumor action because no significant difference between the doses tested was observed. Literature data have shown the in vivo antitumor effects for acridine derivatives [12,13]. However, this is the first time that the mechanism of antitumor action of a spiro-acridine compound has been described.

**Figure 2:**
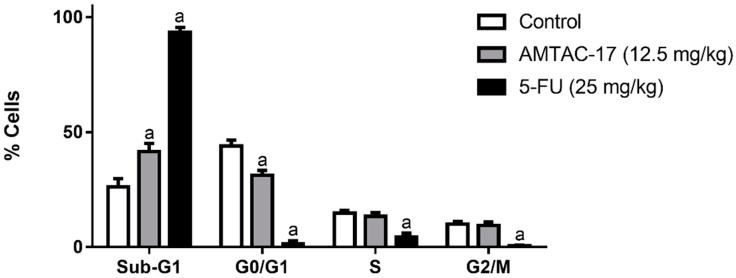
Effects of AMTAC-17 and 5-FU on the cell cycle of Ehrlich ascites carcinoma cells. Percentage of Ehrlich cells in phases of the cell cycle after different treatments: 12% Tween-80 solution (control), AMTAC-17 (12.5 mg/kg), and 5-FU (25 mg/kg). Data presented as mean ± SEM of six animals analyzed by ANOVA was followed by the Tukey test. ^a^ *p* < 0.05 compared to the tumor control group.

On cell cycle analysis, AMTAC-17 induced an increase in the sub-G1 peak to 41.48% (*p* < 0.05), associated with a decrease in the G0/G1 phase to 31.30% (*p* < 0.05), when compared to a control group (26.16 and 43.98%, respectively) (Figure 2). Literature have shown that the increase in the sub-G1 peak is indicative of the apoptosis process [14,15]. In addition, previous studies suggested that acridine derivatives could induce cell cycle arrest and apoptosis [16].

**Figure 3:**
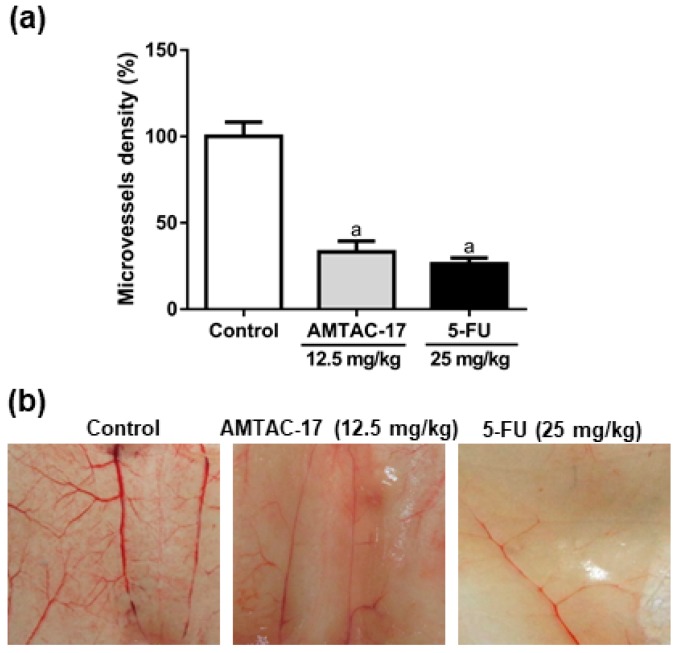
Effects of AMTAC-17 (12.5 mg/kg) and 5-FU (25 mg/kg) on microvessel density (%) in mice transplanted with Ehrlich tumor, after seven days of treatment. (**a**) Microvessel density (%) represents the blood vessel area per field in selected vascularized areas divided by the whole area. (**b**) Representative images of animals’ peritoneum cut into a standard size (1 cm × 1 cm) showing the reduced neovascularization by AMTAC-17 and 5-FU treatments. Data presented as mean ± SEM of six animals analyzed by ANOVA followed by the Tukey test. ^a^ *p* < 0.05 compared to a tumor control group.

AMTAC-17 (12.5 mg/kg) induced a decrease on microvessels’ density (33.16 ± 6.30%) as well as 5-FU (26.26 ± 1.63%), when compared to the tumor control group (100.00 ± 8.27%) (Figure 3).

Tumor cells, together with inflammatory stromal cells, provide bioactive molecules, such as cytokines, chemokines, and other factors to the tumor microenvironment. This creates an immunosuppressive phenotype to favor its proliferation, survival, and angiogenesis [1,17]. The angiogenic process depending on the coordination of many factors is present in the tumor microenvironment and, therefore, understanding the interactions between these components is also relevant for therapeutic strategies against cancer [18,19]. Antiangiogenic therapy is an important target for antitumor drug action, since it prevents the emergence of new blood vessels that support tumor growth and induce metastasis [20]. Our data suggest that the AMTAC-17 mechanism of antitumor action involves an antiangiogenic effect, as previously reported for acridines [12].

**Figure 4:**
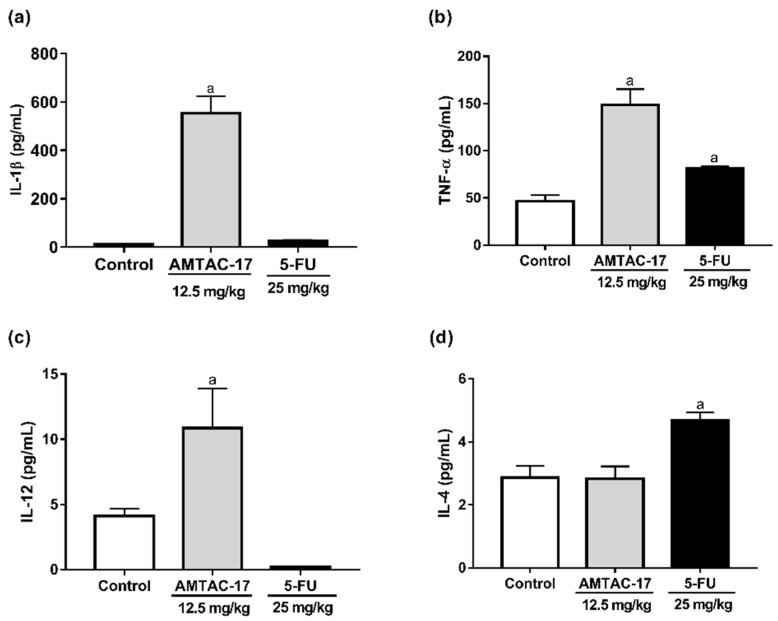
Effect of AMTAC-17 (12.5 mg/kg) and 5-FU (25 mg/kg) on the levels of IL-1β (**a**), TNF-α (**b**), IL-12 (**c**), and IL-4 (**d**) in the peritoneal lavage of Ehrlich ascites carcinoma transplanted mice. Data presented as mean ± SEM of five animals were analyzed by ANOVA, which was followed by the Tukey test. ^a^ *p* < 0.05 compared to the tumor control group.

The AMTAC-17 effects on the inflammatory tumor microenvironment were also investigated. AMTAC-17 (12.5 mg/kg) promoted an increase in proinflammatory cytokine levels, such as IL-1β (553.12 ± 70.57 pg/mL, *p* < 0.05), TNF-α (148.53 ± 16.67 pg/mL, *p* < 0.05), and IL-12 (10.86 ± 3.03 pg/mL, *p* < 0.05), when compared to the control (10.08 ± 3.57 pg/mL, 46.59 ± 6.43 pg/mL, and 4.12 ± 0.84 pg/mL, respectively). No significant change was observed for IL-4 levels after AMTAC-17 treatment. For 5-FU, increases in TNF-α (81.14 ± 2.52 pg/mL, *p* < 0.05) and IL-4 (4.67 ± 0.26 pg/mL, *p* < 0.05) levels were observed (Figure 4).

IL-12 is known for the antiangiogenic action, which reduces the production of the vascular endothelial growth factor (VEGF) and metalloproteinase-9 [21]. Then, the AMTAC-17 effect on microvessels density may be dependent on IL-12 antiangiogenic action. Additionally, IL-12, IL-1β, and TNF-α represent the Th1 cytokines profile, which is known for the cytotoxic effect against cancer cells [22,23]. This effect involves macrophage activation to secrete proinflammatory cytokines and produce reactive oxygen species and nitric oxide [24]. Therefore, the antitumoral effect of AMTAC-17 was also associated with immunomodulation of the tumor microenvironment to induce cytotoxic effects against tumor cells.

In conclusion, AMTAC-17, which is a novel spiro-acridine compound, exerts in vivo antitumor activity by modulation angiogenesis and induces Th1-biased immunomodulation.

## 3. Materials and Methods

### 3.1. Synthesis Methodology

The spiro-acridine compound AMTAC-17 was synthesized at the Drug Development and Synthesis Laboratory (LDSF) of the State University of Paraíba, under the responsibility of Dr. Ricardo Olimpio de Moura, according to the methodologies described previously [8,9]. It was duly characterized by spectroscopic techniques of 1H Nuclear Hydrogen Nuclear Magnetic Resonance on 500 MHz Bruker Avance, Ultrashield^®^ spectrometers. Infrared (IR) (Recife, Pernambuco, Brazil) was obtained by the Attenuated Total Reflectance (ATR) technique in the range of 4000 to 650 cm^−1^ in 63 Perkin Elmer, Spectrum 400 equipment. The results were interpreted by graphs plotted in Origin software 8.0. In addition, mass spectrometry was performed by the MALDI-TOF Autoflex III apparatus technique (Bruker Daltonics, Billerica, MA, USA). This unambiguously characterizes the said structure (Figures S1–S5).

AMTAC-17: Yellow poder - C_27_H_24_N_4_O_4_, W.M = 468.5040 g/mol, rdt = 81.28%, F.F = 237–239 °C theoretical logP = 3.14. RMN1H d6 500MHz. δ = 9.89 (s, 1H, NH), 8.63 (s, 1H, CH), 8.40 (s, 1H, N = CH), 7.29 (m, 2H, spiro-acridine), 7.04 (m, 4H, spiro-acridine), 6.86 (m, 2H, spiro-acridine), 6.70 (s, 2H, phenyl), 3.71 (s, 6H, OCH3), 3.63 (s, 3H, OCH3), 13C d6 100MHz δ = 161.6 (C, C = O), 160.8 (C, C = C), 148.8 (C, Ar), 139.2 (C, Ar), 137.2 (C, Ar), 131.7 (C, Ar), 130.2 (C, Ar), 127.3 (C, Ar), 124.1 (C, Ar), 123.1 (C, Ar), 122.1 (C, Ar), 120.6 (C, Ar), 115.4 (C, Ar), 112.9 (C, CN), 111.8 (C, Ar), 110.0 (adjacent CN), 69.7 (C, sp3). IVcm^−1^ 3350 (N-H amida, 2235 (CN), 1708 (C = O), 1611 e 1479 (C = C phenyl), 1233 e 1130 (Ph-OCH3) 884 e 741 (C-H phenyl), M^−1^ = 467.0608 (found).

### 3.2. Animals

Swiss albino mice (*Mus musculus*), which were females (28–32 g), obtained from the Dr. Thomas George Bioterium (Research Institute in Drugs and Medicines/Federal University of Paraíba, Paraíba, Brazil) were used. The animals were kept under controlled conditions (21 ± 1 °C, 12-h on/12-h off light-dark cycle). The Ethical Committee on the Use of Animals (CEUA)/UFPB (n°. 163/2015) previously approved all procedures.

### 3.3. Acute Non-Clinical Toxicity Assay

The acute toxicity assay was performed, according to the Guideline for Testing of Chemicals *n*. 423 of the Organisation for Economic Co-operation and Development (OECD) (2001). Mice (*n* = 3/group) were subjected to a single dose of 2000 mg/kg AMTAC-17, intraperitoneally (i.p.). The control group was administered to the vehicle alone (12% (*v*/*v*) Tween-80 in saline). The dose responsible for the death of 50% of the experimental animals (LD_50_) was estimated.

### 3.4. In Vivo Antitumor Activity

The Ehrlich carcinoma cell line was generously provided by the Pharmacology and Toxicology Division, CPQBA, UNICAMP (Paulínia, Brazil). The cells were maintained in the peritoneal cavities of Swiss mice in the Dr. Thomas George Bioterium (Research Institute in Drugs and Medicines/Federal University of Paraíba, Paraíba, Brazil).

Five to seven-day-old Ehrlich tumor cells, 0.5 mL (2 × 10^6^ cells/mL), were implanted in the peritoneal cavity of the mice (*n* = 6/group). During the next day, AMTAC-17 (12.5, 25, or 50 mg/kg) was administered for seven consecutive days (i.p.). 5-Fluorouracil (5-FU, 25 mg/kg) was used as a positive control. The tumor control group was treated with 12% (*v*/*v*) Tween-80 in saline. On the eighth day, the animals were anesthetized with ketamine (100 mg/kg) and xylazine hydrochloride (16 mg/kg), and then euthanized [25]. Total viable cancer cells were obtained as the product of the tumor volume (mL) by cell viability (number of cells × 10^6^/mL).

### 3.5. Peritoneal Angiogenesis

Animal’s peritoneum of the tumor control, 12.5 mg/kg AMTAC-17, and 25 mg/kg 5-FU groups was cut open and the inner lining of the peritoneal cavity was examined and photographed. Microvessel density was calculated as the blood vessel area per field in selected vascularized areas divided by the whole area, using AVSOFT^®^ software [26].

### 3.6. Cell Cycle Analyses

Cells from ascitic fluid (1 × 10^6^ cells) of the tumor control, 12.5 mg/kg AMTAC-17, and 25 mg/kg 5-FU groups were harvested, gently fixed by using 70% ice-cold ethanol, and frozen overnight (−20 °C). Samples were harvested by centrifugation (400× *g*, 10 min), and then incubated with RNAse (0.1 mg/mL) and propidium iodide (PI) (0.05 mg/mL) (Sigma Aldrich, St. Louis, MO, USA) (37 °C, 30 min) [22] with modifications. The DNA content was analyzed by flow cytometry (BD FACSCantoTM II, Woburn, MA, USA) and 10,000 events were acquired. After cell debris removal, a gate was placed on PE 585/42 nm-W (width) vs. the PE 585/42-A (area) graph to remove doublets on the right of single cell analysis. The gate with single cells was used to analyze the cell cycle as a histogram on PE 585/42-A. Flowing software version 2.0 was used to analyze the data.

### 3.7. Quantification of Cytokines

The determination of IL-1β, IL-12, TNF-α, and IL-4 cytokine levels was performed using the ascitic fluid collected from the peritoneal cavity of the tumor control, 12.5 mg/kg AMTAC-17, and 25 mg/kg 5-FU groups, using an ELISA kit following the manufacturer’s instructions (ELISA – BIOSCIENCE, Inc. Science Center Drive, San Diego, CA, USA). Optical density was read at 450 nm using a microplate spectrophotometer (Microplate reader BioTek Instruments, Sinergy HT, Winooski, VT, USA).

### 3.8. Statistical Analysis

Data are presented as mean ± standard error of mean (SEM) and analyzed by the GraphPad Prism 7.0 software (GraphPad Software Inc., San Diego, CA, USA). The differences between experimental groups were compared by analysis of variance (ANOVA). Lastly, the Tukey’s test (*p* < 0.05) was performed.

## Supplementary Materials

The following are available online at https://www.mdpi.com/1420-3049/25/1/29/s1, Figure S1: Hydrogen Nuclear Magnetic Resonance Spectrum of AMTAC-17 compound, Figure S2: Expanded Hydrogen Nuclear Magnetic Resonance Spectrum of AMTAC-17 compound in the aromatic region, Figure S3: Carbon Nuclear Magnetic Resonance Spectrum 13 (DEPTQ) of AMTAC-17 compound, Figure S4: Infrared Spectrum of AMTAC-17 compound, Figure S5: Mass spectrum of AMTAC-17 compound.
