# Supplementary material for: Antitumor Effect of a Novel Spiro-Acridine Compound is Associated with Up-Regulation of Th1-Type Responses and Antiangiogenic Action
Source: Molecules. 2019 Dec 20;25(1):29. doi: 10.3390/molecules25010029 (PMC6982894; doi:10.3390/molecules25010029)

Supplementary files

Figure 01 - Hydrogen Nuclear Magnetic Resonance Spectrum of AMTAC-17 Compound.

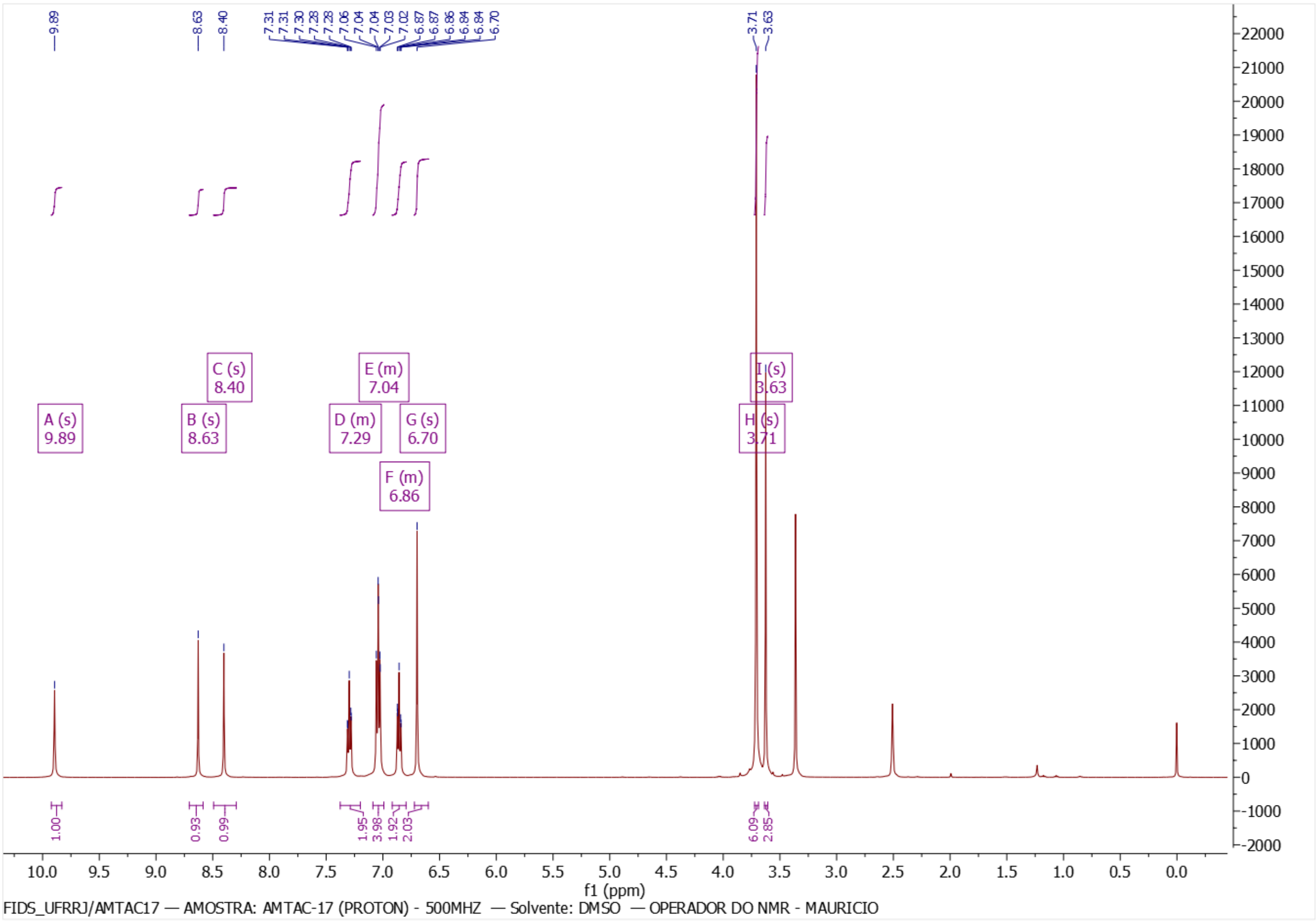

Figure 02 - Expanded Hydrogen Nuclear Magnetic Resonance Spectrum of AMTAC-17 Compound in the aromatic region

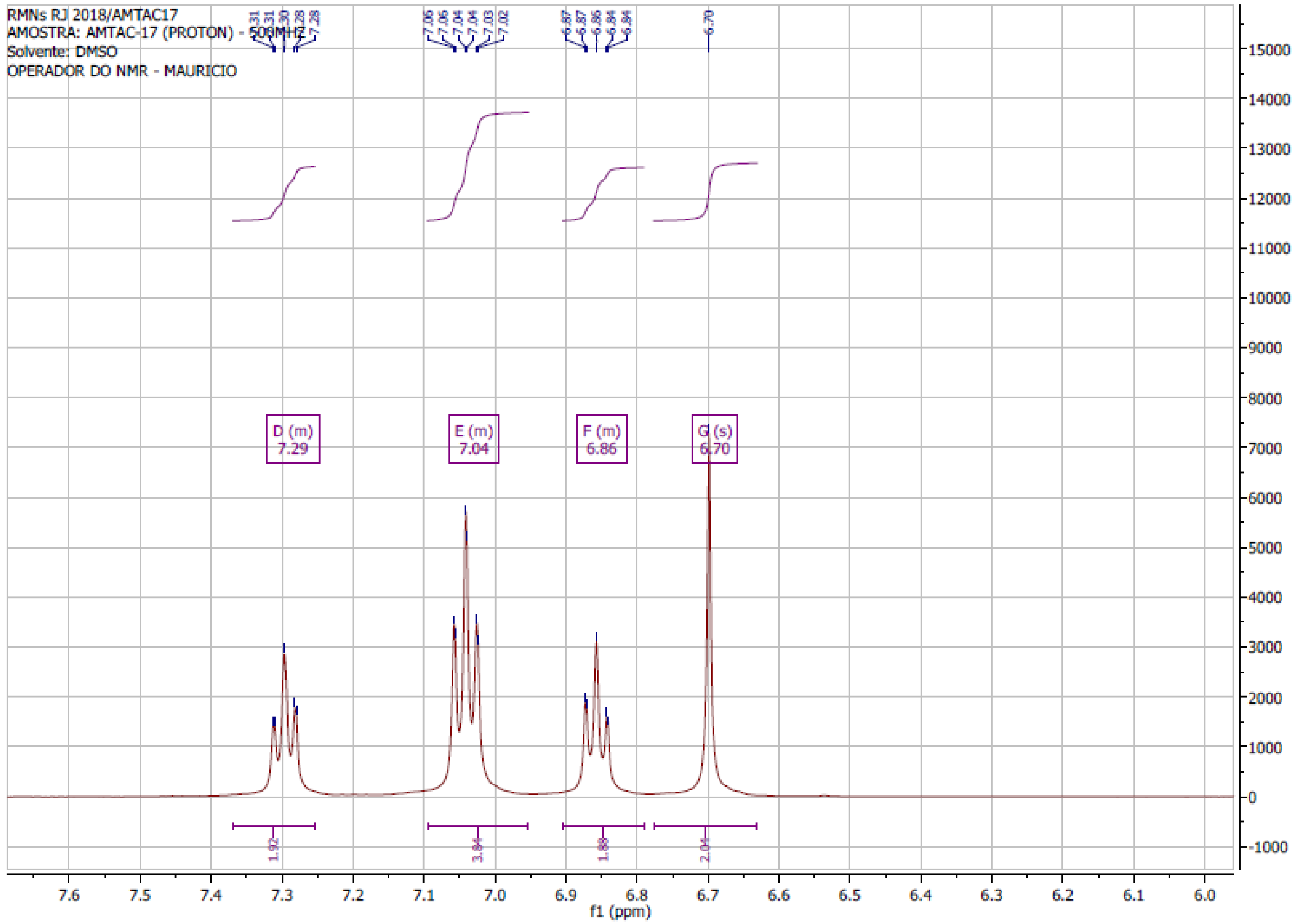

Figure 03 - Carbon Nuclear Magnetic Resonance Spectrum 13 (DEPTQ) of AMTAC-17 Compound.

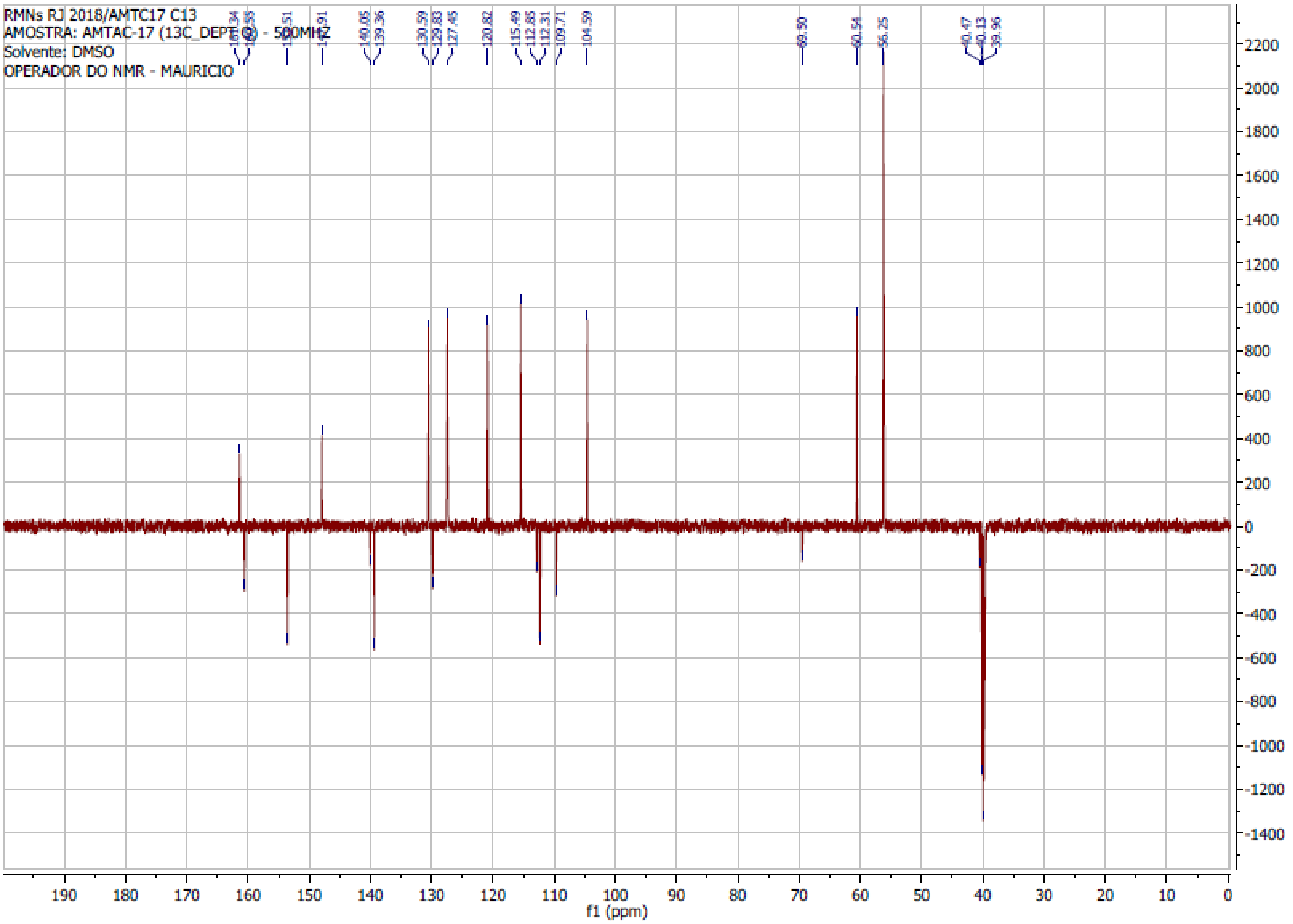

Figure 04 - Infrared Spectrum of AMTAC-17 Compound

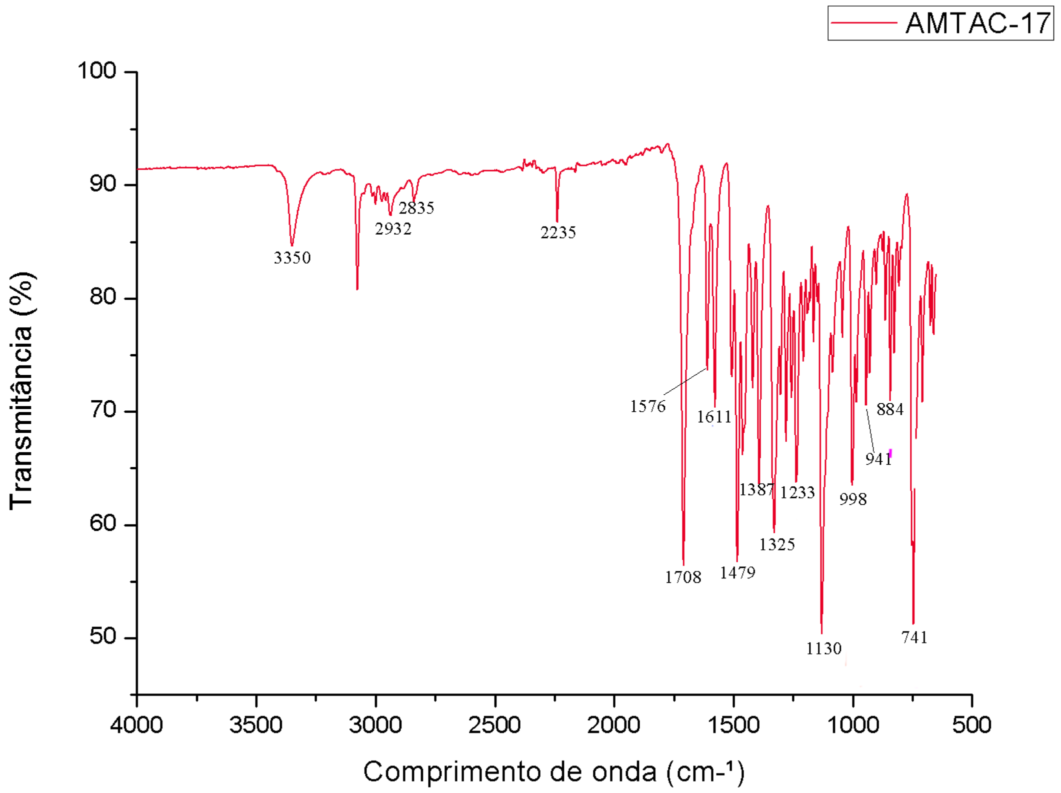

Figure 05 - Mass spectrum of compound AMTAC-17.

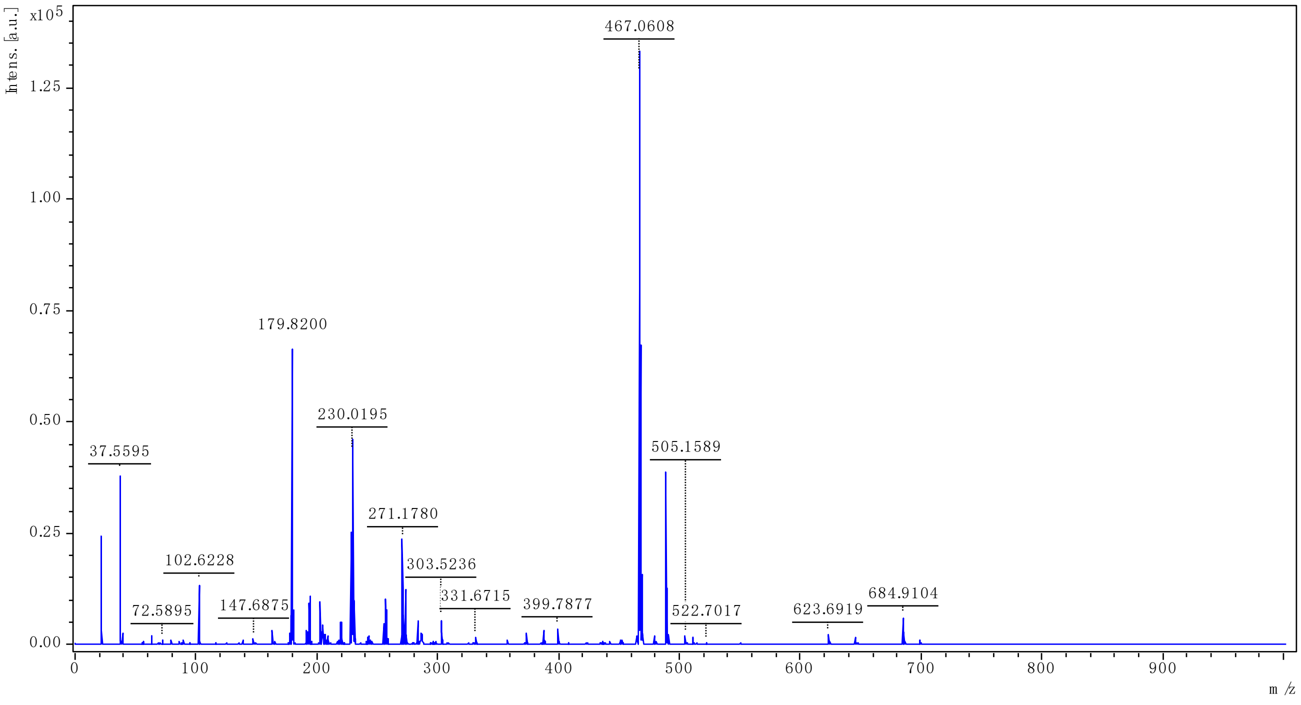

Supplement: Supplementary file 1 [file molecules-25-00029-s001.pdf]
